# The effect of graft application and allopurinol treatment on calvarial bone defect in rats^1^

**DOI:** 10.1590/s0102-865020190030000006

**Published:** 2019-03-18

**Authors:** Nihat Laçin, Bozan Serhat İzol, Ebru Gökalp Özkorkmaz, Buşra Deveci, Mehmet Cudi Tuncer

**Affiliations:** I PhD, Assistant Professor, Department of Oral and Maxillofacial Surgery, Faculty of Dentistry, University of Katip Çelebi, İzmir, Turkey. Technical procedures, manuscript preparation and writing, final approval.; II PhD, Research Assistant, Department of Periodontology, Faculty of Dentistry, University of Bingöl, Bingöl, Turkey. Technical procedures, manuscript preparation and writing, final approval.; III PhD, Assistant Professor, Department of Histology and Embryology, Faculty of Medicine, Dicle University, Diyarbakır, Turkey. Technical procedures, histopathological examinations, manuscript preparation and writing, final approval.; IV PhD, Research Assistant, Department of Periodontology, Faculty of Dentistry, University of Dicle, Diyarbakir, Turkey. Technical procedures, manuscript preparation and writing, final approval.; V PhD, Professor, Department of Anatomy, Faculty of Medicine, Dicle University, Diyarbakır, Turkey. Technical procedures, histopathological examinations, manuscript preparation and writing, final approval.

**Keywords:** Osteonectin, Osteopontin, Allopurinol, Skull, Rats

## Abstract

**Purpose:**

To investigate the effects of allopurinol administration on osteoinductive reaction and bone development with graft material.

**Methods:**

Thirty-six Wistar albino rats were divided into 3 groups. In the control group, calvarial bone defect was only created without any treatment. In the Defect + Graft group, allograft treatment was performed by forming 8 mm calvarial bone defect. In the Defect + Graft + Allopurinol group, alloplastic bone graft was placed in the calvarial bone defect and then, allopurinol (50 mg/kg/day) treatment was intraperitoneally applied for 28 days.

**Results:**

Histopathological examination revealed inflammation, congestion in the vessels, and an increase in osteoclast cells in the defect area. We also observed that new osteocyte cells, increase in connective tissue fibers, and new bone trabeculae. Osteopontin expression was positive in osteoblast cells and lacunated osteocyte cells were located in the periphery of the new bone trabeculae. Osteopontin expression was also positive in osteoblasts and osteocytes cells of new bone trabeculae in the graft site.

**Conclusion:**

It has been shown that allopurinol treatment in rat calvaria defects may induce osteoblastic activity, matrix development, mature bone cell formation and new bone formation when used with autogenous grafts.

## Introduction

 Bone defects in the maxillofacial region can appear after trauma, infection, bone tumours or cysts and orthognathic surgical procedures. While small defects in the bone can be repaired by natural bone healing processes, large defects require grafts and implants, using various materials^1^. Calvarial critical dimension defects have been widely used to evaluate bone regenerative materials. In these defects, it is important to maintain a suitable area due to the competition between the surrounding soft tissues and bone formation into the defect by using barrier membranes^2^. For the rat calvarial defect, 8 mm is generally accepted to be of critical size^3^.

Cortical grafts provide a durable and rigid structure, but they have no ability to increase osteogenesis in the experimental and human studies. The primary advantage of cancellous bone and bone marrow is they are able to significantly enhance osteogenesis. These abilities depend on the fact that they have viable cells that can transform into osteoblasts as well as those that induce osteogenesis. The only known disadvantage of these grafts is they can’t provide mechanical stability^4^ ^,^ ^5^. Allografts are bone tissues obtained from genetically different individuals but sharing properties of same species with donor. Fresh frozen bone can be classified as frozen dried bone and demineralized bone matrix. Due to the limited availability of autografts, undesirable features of allografts and xenografts such as the risk of disease transfer, researchers have now been focused on synthetically graft materials that are produced for use in bone defects^6^ ^,^ ^7^. Alloplastic bone grafts are synthetic, inorganic, biocompatible, and bioactive bone substitutions which are believed to repair bone defects through osteoconduction. Allografts are used freeze-dried demineralized bone allograft (FDBA) and demineralized freeze-dried bone allograft form known as (DFDBA)^8^. FDBA provides an osteoconductive effect to bone regeneration while DFDBA is to allow an osteoconductive surface while maintaining the additional benefit of its functioning as a source for the osteoinductive factors^9^. 

 Different methods for the treatment of calvarial bone defects have been used in experimental studies on rats. However, the number of experimental studies are quite limited^10^ ^-^ ^17^. Diomede *et al.* ^10^ found that membrane scaffold evolution, available biomaterial with a high consistency dense collagen fiber derived from equine mesenchymal tissue, enriched with human periodontal ligament stem cells and conditioned medium showed a higher osteogenic ability compared with the other complexes, being able to almost completely repair the rat calvarial defect^10^. The findings of Bizenjima *et al.* ^11^ suggested that the application of the lactide-co-glycolide-coated ß-tricalcium phosphate could promote bone regeneration to similar extent as the pure ß-tricalcium phosphate material in a rat calvarial bone model^11^. Similarly, Wang *et al.* ^12^ suggested that lactic-co-glycolic acid- alendronate may be a potential bone graft substitute to enhance bone repair in a rat femoral bone defect model^12^. And also, it was reported that alendronate enhanced the new bone formation by autogenous bone graft in the rat calvarial defect model^13^. Fu *et al.* ^14^ reported that combination of calcium sulfate and simvastatin-controlled release microspheres enhances bone repair in critical-sized rat calvarial bone defects^14^. In addition, it was reported that 0.1 mg simvastatin was the optimal dose for stimulation of the maximum bone regeneration in rat calvarial defects without inducing inflammation^15^. They suggested that zoledronic acid treatment, a drug used to treat bone diseases, improved bone formation in calvarial bone formation^16^. In rats with diabetes with calvarial defect model, gaseous ozone administration accelerated xenograft resorption and increased bone regeneration, especially in early stages of bone healing^17^. Similar to these calvarial bone defect models in rats, the effect of graft application with allopurinol treatment were investigated in our study.

 Allopurinol (1,5-dihydro-4Н-pyrazole (3,4-d) pyrimidine-4-one) has been used to treat hyperuricemia^18^, and gout^19^. The most common form of inflammatory arthritis with relatively minor adverse effects. Beneficial effects of allopurinol, or its more stable metabolite oxypurinol, are evidenced in the case of vascular injury, inflammation^20^, heart failure^21^, ischemic heart disease^22^ ^,^ ^23^, and also myocardial protection during cardiac or aortic surgery or post-ischemic reperfusion^24^. Allopurinol has been shown to reduce bone resorption^25^ and promote bone formation^26^.

Osteonectin is a glycoprotein synthesized by cells of an osteoblastic lineage that is abundantly expressed in bones undergoing active remodeling. It binds hydroxyapatite, calcium, and type I collagen, and inhibits mineralization in vitro^27^. Osteonectin helps to connect bone mineral and collagen fibrils to each other. Meanwhile, estrogens increase the differentiation of osteoblast cells and stimulate bone matrix mineralization, and arrange the expression of noncollagenous proteins such as type I collagen and osteopontin, osteocalcin, osteonectin, and the like^28^.

 Osteopontin (OPN) is one of the major non-collagen proteins in extracellular bone matrix, and it plays a role in osteoclast-mediated bone resorption^29^. Osteopontin is expressed by various human cell types in several tissues, including bone, dentin, cement, cartilage, kidney, brain, vascular tissue, and epithelial tissue^30^. OPN is produced by osteoclasts as well as differentiated osteoblasts and osteocytes and plays a role in resorption along with the formation, migration, and attachment of osteoclasts^31^. It was reported that osteopontin acts as a proinflammatory cytokine and plays an important role in regulating the inflammatory process^32^. Baloglu *et al.* ^33^ showed that osteopontin expression was positive in fibroblast and inflammatory cells in bone tissue resulting from estrogen deficiency after ovariectomy.They found that bone tissue was a sign of bone metabolism in the mRNA of osteocytes^33^.

The aim of this study was to investigate the effects of allopurinol administration on calvarial bone defects with graft material whether it is acting in osteoinductive reaction and bone development.

## Methods

 Every single surgical methodology and the consequent care and healing of the animals utilized as a part of this investigation were in strict understanding with the National Institutes of Health (NIH Publications No. 85-23, revised 1985) rules for animal care. This study was approved by the Ethics Committee for Animal Experimentation of the Faculty of Medicine at Dicle University,Turkey. 

In this study, 36 Wistar male rats weighing 280-300 grams were used. The rats were housed individually in suitable cages, at a temperature of 22 ± 2ºC and in 12 hours of dark, 12 hours of light. Animals were fed with standard laboratory food and water. All rats toward the finish of the analysis were healthy and no distinction in nourishment/water consumption and body weight pick up amongst experimental and control rats were noticed.

###  Calvarial defect model 

#### Sedation and surgical procedure

The animals were anesthetized with intraperitoneally 3 mg/kg xylazine (Rompun 2%; Bayer) and 90 mg/kg Ketamine HCl (Ketalar; Eczacıbası-Warner Lambert). Skin was incised to open frontal bone. A periosteal flap was removed with a thin elevator. Surgical sites were exposed with an incision through the skin and the periosteum at the midline of the calvaria. The periosteal flap was removed with a thin periosteal elevator and a specially designed Trephine Bur created a circular full-thickness bone defect with a diameter of 0.8 cm on the midline. The material used in our study was Biograft^®^ HT (IFGL Bio Ceramics) which contains 40% β-Tri Calcium Phosphate with 60% porous biphasic synthetic Hydroxyapatite. This material is an alloplast with granule size of 350-500 µm with osteoconductive properties. So, alloplastic material (Biograft-HT^®^) was placed in defect area in group 2 and group 3. Subcutaneous tissue was sealed with 6/0 vicryl suture and skin was allowed to heal.

 For allopurinol treatment, allopurinol in powder form (Sigma-Aldrich, St. Louis, MO) was dissolved in saline, and 2 M NaOH was added to generate a final pH of approximately 10,5^34^. The allopurinol solution was injected intraperitoneally for 4 weeks at a concentration of 50 mg/kg of body weight. At the end of the study, animals were anesthetized with intraperitoneally 3 mg/kg xylazine and 90 mg/kg Ketamine HCl, then all animals were sacrified by decapitation. The skin, as well as all of the soft tissues surrounding the calvarial bone were removed. The samples were fixed with 10% neutral buffered formalin solution and decalcified with 5% EDTA (Ethylenedaiminetetraacetic acid). After rinsing with tap water, the samples were dehydrated in increasing concentrations of ethanol and embedded in paraffin. Tissue sections of 4-6 µm thickness were prepared in the transverse plane and stained using Hematoxylin-eosin for light microscopy examination.

Three groups (12 rats per group) were arranged as below:

1. **Control group:** 8 mm calvarial bone defect was sutured without any treatment. The subjects were sacrificed at the end of the 4^th^ week;
2. **Defect + Graft group:** 8 mm calvarial bone defects were created in all rats and then alloplastic bone grafts were applied to the defect. The subjects were sacrificed at the end of the 4^th^ week;
3. **Defect + Graft + Allopurinol group:** Alloplastic bone graft was placed in the calvarial bone defect and then, allopurinol (50 mg/kg/day) treatment was intraperitoneally applied for 28 days. Rats were sacrificed at the end of the 4^th^ week.

###  Immunohistochemical staining 

 Antigen retrieval was done in microwave (Bosch^®^, 700 watt) for 3min x90^o^C. They were subjected to a heating process in a microwave oven at 700 watts in a citrate buffer (pH 6) solution for proteolysis. Sections were washed in 3x5 min PBS and incubated with hydrogen peroxide [K-40677109 ,64271 Hydrogen peroxide (H_2_O_2_) Dortmudt+Germany, MERCK] (3ml %30 Hydrogen peroxide (H_2_O_2_) + 27ml methanol) for 15 min. Sections were washed in 3x5 min PBS min and blocked with Ultra V Block (lot: PHL150128, Thermo Fischer, Fremont, CA, USA) for 8 min. After draining, primary antibodies were directly applied to sections distinctly Osteonectin (SPARC), Catalog #:33-5500, 1:100, Thermo Fischer, Fremont, CA, USA, Osteopontin monoclonal antibody 1:100, (MA5-17180) Thermo Fischer, Fremont, CA, USA. Sections were incubated and left overnight at 4^o^C. Sections were washed in 3x5 min PBS and then incubated with Biotinylated Secondary Antibody (lot: PHL150128, Thermo Fischer, Fremont, CA, USA) for 20 min. After washing with PBS, Streptavidin Peroxidase (lot: PHL150128, Thermo Fischer, Fremont, CA, USA) was applied to sections for 15 min. Sections were washed in 3x5 min PBS and DAB (lot: HD36221, Thermo Fischer, Fremont, CA, USA) were applied to sections up to 10 min. Slides showing reaction was stopped in PBS. Counter staining was done with Harris’s Haematoxylin for 45 sec, dehydrated through ascending alcohol and cleared in xylene. Product Number: HHS32 *SIGMA,* Hematoxylin Solution, Harris Modified, Sigma-Aldrich, 3050 Spruce Street, Saint Louis, MO 63103, USA. Slides were mounted with Entellan^®^ (lot: 107961, Sigma-Aldrich, St. Louis, MO, United States) and examined under light microscope (Zeiss, Germany). 

###  Semi-quantitative score of histopathologic parameters 

 Semi-quantitative score was determined by examining osteoblast cells, osteocyte cells, inflammation, congestion in blood vessels, new bone formation, and osteoclast cells in the bone tissue in 15 different regions within the microscope field, and 10 cells counted in each area. Similar semi-quantitative methods have been used in histochemical studies of bone tissue^35^ ^-^ ^37^.

###  Statistical analysis 

 Statistics and analyzes were performed using the SPSS 22.0 for Windows computer package program. In the analysis of the data, Kruskall-Wallis and Mann-Whitney U non-parametric statistical tests were used in the intergroup comparisons depending on the variables and the results were given as the mean ± standard deviation and mean rank. And, the results were considered statistically significant for P=0 with Kruskal-Wallis test and P < 0.05 with Mann-Whitney U test.

## Result

**Figure 1:**
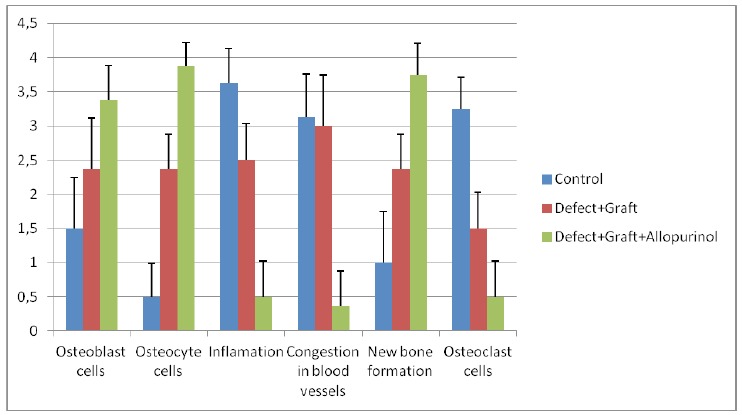
Graphic showing histopathological difference in control and experimental groups. The quantification of all parameters: **0:** no change, **1:** too week, **2:** week, **3:** middle, **4:** strong. (Scoring was determined by examining histological parameters in 15 different regions within the microscope field, and 10 cells counted in each area).

 The histopathological results of the present study were evaluated under light microscope. We compared histopathological findings in the control and other experimental groups (Table 1, Fig. 1).

**Table 1:** Histopathological scoring of control and experimental groups. Data are expressed as the mean ± standard deviation and mean rank (*P=0 with Kruskal-Wallis test, **P < 0.05 with Mann-Whitney U test, * and ** statistically significant result).

Parameter	Groups	n	Mean±SD	Mean Rank	Kruskal-Wallis Test value	Mann-Whitney U comparisons for groups (p<0.05
Osteoblast cells	*(1) Control*	*12*	1.50±0.75	*6.50*	*13.762* *P=0.001	**(2) **(3)
	*(2) Defect+Graft*	*12*	2.37±0.74	*12.06*		**(1) **(3)
	*(3) Defect+Graft+Allopurinol*	*12*	3.37±0.51	*18.94*		**(1) **(2)
Osteocyte cells	*(1) Control*	*12*	0.5±0.52	*4.50*	*20.978* *P=0	**(2) **(3)
	*(2) Defect+Graft*	*12*	2.37±0.51	*12.69*		**(1) **(3)
	*(3) Defect+Graft+Allopurinol*	*12*	3.87±0.35	*20.31*		**(1) **(2)
Inflammation	*(1) Control*	*12*	3.62±0.51	*19.75*	*19.645* *P=0	**(2) **(3)
	*(2) Defect+Graft*	*12*	2.50±0.53	*13.25*		**(1) **(3)
	*(3) Defect+Graft+Allopurinol*	*12*	0.50±0.53	*4.50*		**(1) **(2)
Congestion in blood vessels	*(1) Control*	*12*	3.12±0.64	*16.88*	*16.541* *P=0	**(3)
	*(2) Defect+Graft*	*12*	3.00±0.75	*16.12*		**(3)
	*(3) Defect+Graft+Allopurinol*	*12*	0.37±0.51	*4.50*		**(1) **(2)
New bone formation	*(1) Control*	*12*	1.00±0.75	*5.12*	*19.024* *P=0	**(2) **(3)
	*(2) Defect+Graft*	*12*	2.37±0.51	*12.25*		**(1) **(3)
	*(3) Defect+Graft+Allopurinol*	*12*	3.75±0.46	*20.12*		**(1) **(2)
Osteoclast cells	*(1) Control*	*12*	3.25±0.46	*20.50*	*19.422* *P=0	**(2) **(3)
	*(2) Defect+Graft*	*12*	1.50±0.53	*11.50*		**(1) **(3)
	*(3) Defect+Graft+Allopurinol*	*12*	0.50±0.53	*5.50*		**(1) **(2)

###  Histological analysis 

#### 1. Defect group

**Figure 2:**
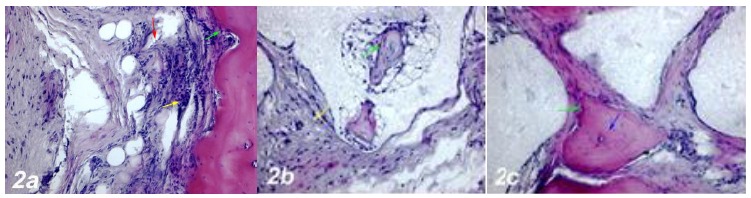
**a. Haematoxylin-eosin staining (Control group).** Dense inflammatory cell infiltration (*yellow arrow*), dilatation and congestion in the blood vessels (*red arrow*), an increase in osteoclast cells, degeneration and apoptotic changes in osteoblast cells (*green arrow*). **b. Haematoxylin-eosin staining (Defect + Graft group).** An incresea in osteoblast (green arrow), and osteocyte cells in trabecular bone around graft area, reduction of inflammation in connective tissue (yellow arrow). **c. Haematoxylin-eosin staining (Defects + Graft + Allopurinol group).** A significant increase in osteoinductive effect of osteoblasts (*green arrow*), an increase in the number of osteocyte cells in bone trabeculae (*blue arrow*). Scale bar = 50 μm.

Dense inflammatory cell infiltration, dilatation and obstruction in the blood vessels, increased osteoclast cells and necrotic changes were observed in the defect area near the calvarial bone. Degeneration of osteoblast cells and apoptotic changes in osteocyte cells were also observed (Fig. 2a). 

#### 2. Defect + Graft group

In the area of graft, mitotic activity and matrix development were observed in osteocyte and osteoblast cells in small bone trabeculae Histological sections showed decreased collagen fiber growth and connective tissue. And, osteoinductive effect of new bone trabeculae was observed to mature (Fig. 2b). Çalışmamızda greft grubunda osteoindüktif etki artarak küçük kemik trabekülleri oluşmaya başlamıştır.Yeni kemik oluşumunun ilk belirtisi oluşmuştur.

#### 3. Defects + Graft + Allopurinol group

An increase in collagen fibers was observed in connective tissue cells and around the graft site. A significant increase in the osteoinductive effect of osteoblasts has led to an increase in the number of osteocyte cells in bone trabeculae. An increase in osteogenic matrix formation and a decrease in osteoclastic activity were also observed in inflammatory cells (Fig. 2c). In this group, new bone formation increased significantly.

###  Immunohistochemical examinations 

#### 1. Defect group 

**Figure 3:**
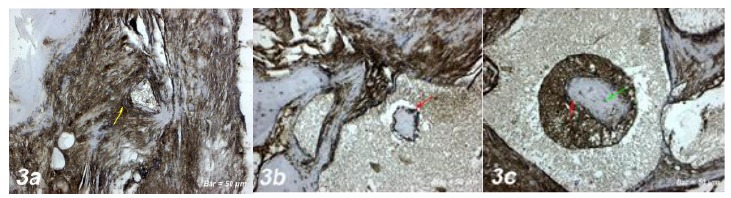
**a. Osteonectin immunostaining (Control group).** Positive osteonectin expression was observed in inflammatory cells, collagen fibers and osteoclastic cells around the blood vessels within the defect area (*yellow arrow*). **b. Osteonectin immunostaining (Defect+ Graft group).** An increase in connective tissue fibers and fibroblast cells around the graft area. Positive osteonectin expression in osteoblast cells (*red arrow*), and osteoclast cells. **c. Osteonectin immunostaining (Defects + Graft + Allopurinol group).** Positive osteonectin expression in osteoblast cells (*red arrow*), and osteocyte cells (*green arrow*) located in the periphery of the new bone trabeculae. Scale bar = 50 μm.

**Figure 4:**
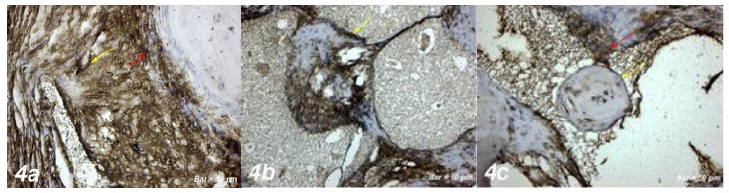
**a. Osteopontin immunostaining (Control group).** Positive osteopontin reaction in fibroblasts and collagen fibers around dilated blood vessels (*yellow arrow*). Negative osteopontin expression in osteoblast cells, positive osteopontin expression in osteoclast cells and inflammatory cells within the defect area (*red arrow*). **b. Osteopontin immunostaining (Defect+ Graft group).** Osteogenetic matrix development in small bone, positive osteopontin expression in osteoblast cells (*yellow arrow*), and osteocyte cells. **c. Osteopontin immunostaining (Defects + Graft + Allopurinol group).** Positive osteopontin expression in osteoblast cells (*yellow arrow*), and osteocyte cells in the periphery of the new bone trabeculae (*red arrow*). Scale bar = 50 μm.

Positive osteonectin expression was observed in inflammatory cells, collagen fibers and osteoclastic cells around the blood vessels within the defect area Figure 3a). A positive osteopontin reaction was observed in fibroblasts and collagen fibers around dilated blood vessels. Negative osteopontin expression was observed in osteoblast cells and positive osteopontin expression was observed in osteoclast cells and inflammatory cells within the defect area (Fig. 4a).

#### 2. Defect + Graft group

An increase was observed in connective tissue fibers and fibroblast cells around the graft area. Osteonectin expression was positive in osteoblast cells and a small number of osteoclast cells (Fig. 3b). Osteogenic matrix development was started in small bone trabeculae in the graft area and osteopontin expression in osteocyte cells was observed. An increase in connective tissue fibers and cellular structures was observed in the graft area. Osteopontin expression showed positive reaction in osteblast cells while osteoclast cells decreased. Osteopontin expression showed a positive reaction with expansion of bone trabeculae and increased osteoid cells. (Fig. 4b).

#### 3. Defect + Graft group + Allopurinol group

An increase in connective tissue fibers and new bone trabeculae was observed in the graft area. Osteonectin expression was positive in osteoblast cells and lacunated osteocyte cells located in the periphery of the new bone trabeculae (Fig. 3c). Osteopontin expression was positive in osteoblasts and osteocytes of new bone trabeculae in connective tissue fibers (Fig. 4c).

## Discussion

 The calvarial bone defect model is appropriate for the examination of maxillary bone regeneration because it has several similarities to the maxillofacial area^38^ ^,^ ^39^. The critical-size rat calvarial defect, compared with other experimental bone defects, is a convenient model for evaluating bone regenerative effects of biomaterials. Research mainly including experimental calvarial defect models are also currently conducted to find out materials with potential, if any, osteopromotive effect in bone repair or regeneration^40^. Healing occurs after regeneration of bone, infiltration of granulation tissue, remodelization of osteogenic cells with proliferation. The following case continues with a cellular activity that begins with an acute inflammatory response. Bone formation depends on the cooperation of various factors such as specific cell types like mesenchymal stem cells and soluble molecules such as osteoclasts, hydroxyapatite, extracellular matrix molecules, cytokines, and bone morphogenetic proteins, hormones, vitamins and various factors such as growth factors have been reported^26^.

Different bone graft materials have been used for bone regeneration, closure of osteotomy openings, and alveolar augmentation in oral and maxillofacial surgeons^41^. Natural coral-derived grafts and synthetic bone graft materials are used in alveolar crest elevation, intra-bone defects, material loss fractures, facial bone defects, orthognathic surgery, and maxillary sinus ground^42^ ^-^ ^44^. Mah *et al.* ^45^ stated that the allograft material could not be increased beyond the control levels of bone formation, but instead the advantage of the implant materials could be included in the healing zone of the material and in filling the defect by rapid bridging of the wound^45^. In our study, an alloplastic graft material consisting of a combination of 350-500 μm-diameter porous biphasic hydroxyapatite granules and β-tricalcium phosphate granules was used. Small bone trabeculae were formed in the graft group by increasing the osteoinductive effect. And, the first sign of new bone formation was formed (Fig. 2b).

Osteonectin is a protein known to be involved in cell-matrix interactions and angiogenesis. The new vessel formation by affecting angiogenesis osteonectin and, it is important for normal ossification. Koparal *et al.* ^16^ were to investigate melatonin application in tibial bone defects. They found that osteonectin expression was prominent in the bone matrix and osteoblasts cells. And on the 14th day, they found that bone mineralization accelerated. Osteonectin is secreted by osteoblasts in bone during bone development and remodeling. Osteonectin helps to connect bone mineral and collagen fibrils to each other^46^. In our study, osteonectin expression was positive in osteoblast cells after graft application (Fig. 3b). In the graft + allopurinol administration, osteonectin protein expression was increased in osteoblast and osteocyte cells, and new bone formation was formed (Fig. 3c).

 Osteopontin (OPN) is a non-collagenous matrix protein. OPN is known to play a role in cell adhesion, migration, survival, and bone remodeling^47^ ^,^ ^48^. Research in animal models indicates that OPN deficiency alters the functionality of multiple cell types, resulting in delayed early vascularization, altered matrix organization, and late bone remodeling^32^. Baloglu *et al.* ^33^ reported that they induced bone matrix development in rats with clomiphene citrate after ovarectomy and induced new bone formation with increased osteonectin and osteopontin expression. Osteopontin has been shown to mediate osteoclast development by mediating cell-cell contact between osteoblastic cells and osteoclast progenitors. Osteopontin has been reported to increase the effect of paracrine cytokines produced by stromal/ osteoblastic cells, thus promoting the proliferation or differentiation of hematopoietic precursors^49^. In our study, osteopontin expression showed positive reaction in osteoblasts in small bone trabeculae after graft application (Fig. 4b). Application of allopurinol with graft resulted in increased osteopontin expression in osteoblast cells and osteocytes in bone trabeculae, and a new bone formation by mineralization with the development of osteoid tissue (Fig. 4b).

Allopurinol is a potential treatment for a range of conditions including chronic heart failure ischemia-reperfusion injury, vascular disease, chronic kidney disease and diabetes^20^. Orriss *et al.* ^26^ demonstrated that allopurinol and oxaquinoline increased osteoblast differentiation and bone formation in vitro but did not affect osteoclast function. They demonstrated by in vitro studies that inhibition of XO activity promotes osteoblast differentiation and leads to increased bone formation. In our study, osteoclastic activity was observed in the group that had calvarial defect and graft treated.In the allopurinol treated group, there was a significant decrease in osteoclast cells in the defect and graft region due to the decrease in inflammation, and the osteoblast cell development increased and the osteocyte cells were spreading and induced new bone formation (Fig. 2c). Miesel *et al.* ^50^ have suggested that antioxidants such as allopurinol, which alter the oxidative burst of phagocytes, inhibit xanthine oxidase and exhibit immunosuppressive effects, show chronic phagocytic hyperactivity in rheumatoid arthritis. Namazi^51^ reported that allopurinol, a competitive xanthine oxidase inhibitor, reduced serum uric acid levels in autoimmune disorders such as rheumatoid arthritis. In one study, allopurinol showed antiinflammatory effect by decreasing Prostoglandin E level which may be useful in delaying the complication of rheumatoid arthritis^52^.

## Conclusion

It has been shown that that allopurinol treatment in rat calvaria defects can induce osteoblastic activity, matrix development, mature bone cell formation and new bone formation by using autogenous grafts.
